# Social Problems at the Church Congress

**Published:** 1907-11-09

**Authors:** 


### SOCIAL PROBLEMS AT THE CHURCH CONGRESS.

"The Church and Poor Relief" was the subject of some interesting papers at the Church Congress. Mrs. Barnett, of Toynbee Hall, who has an unrivalled experience among the poor, protested against the present tendency to institutionalism. The cost is enormous—thirteen million pounds are spent annually in poor relief—and it does not lessen pauperism, for one person in thirty-eight is in receipt of poor relief. Mrs. Barnett pleaded, however, for the State to do its best for the children, and not to let them suffer in order to punish their unworthy parents. Prebendary Carlile, the head of the Church Army, gave more definite advice about the prevention of pauperism. It involved some control of the individual by keeping him hard at work, shielding him from the temptation to drink, and paying him fair wages for his labour. He mentioned a tramp who, being reformed, rose to be a churchwarden, but admitted that this was an exceptional case; but he claimed that if he had any good left in him—and few were absolutely hopeless—the poor man, if taken in hand in time and in the right way, would develop an interest in his work and study with satisfaction the growth of his savings-bank account. For the determined idler and loafer he suggested semi-penal colonies, to which he would be sentenced for three years, or even for indeterminate periods, and where he would be compelled to work. For even repeated begging he would send the beggar to these colonies, where he believed the more hopeful among the colonists would soon get to like work. The others might require to remain there for life. As in the Belgian colonies, there would be, Prebendary Carlile thought, no need to exercise special supervision to prevent colonists escaping. If they could not make an honest living they would soon be sent back, and if they did they had a right to their liberty. He spoke of the usefulness of the Church Army homes for men who were going to leave the penal colonies. They might have considerable difficulty in obtaining work, their industrial record not being good, and in the home they could remain until they found work or it was found for them, while still keeping up their new habit of industry.

The Rev. T. G. Longley, vicar of St. Paul's, Southwark, told of his experience of six years' work in "the blackest of black streets." He deprecated the close combination of spiritual ministrations and temporal relief, which he characterised as little less than religious bribery, and advised the formation in each parish of an undenominational committee, who would give adequate relief where it was needed, and could also advise as to the best help that could be given, for he pointed out how often the poor ask for one sort of assistance when quite another kind is required. He recommended the teaching of applicants to pay what they could for what was given to them, and showed that even in his poor district of London this had been successful. Finally he dwelt upon the importance of thorough investigation of each case, so that those who wanted to help might get at the real cause of the trouble, of which the immediate necessity was often only a symptom. He pleaded for practical common sense in charitable work. "It would be well," he said, "for us all to organise our charities on a business basis with the same care and attention to detail as a city man would regard as imperative in his business, and the result would be a healthier tone among many of our poor, and also in no slight measure a removal of some of those hindrances which at present made difficult the progress of the Kingdom of God." At a time when so many criticisms are being levelled at the national Church, it is good to see in how spiritual, yet practical, a way its clergy deal with the problems of the day.

